# Anti-Inflammatory Effects of Novel Probiotic *Lactobacillus rhamnosus* RL-H3-005 and *Pedicoccus acidilactici* RP-H3-006: In Vivo and In Vitro Evidence

**DOI:** 10.3390/foods13223676

**Published:** 2024-11-18

**Authors:** Shugang Li, Yixuan Li, Donglin Sui, Qingyu Ren, Chunqing Ai, Mingxin Li, Shouhao Zhao, Huan Li, Shuang Song, Xiaomeng Ren

**Affiliations:** Collaborative Innovation Center of Seafood Deep Processing, National Engineering Research Center of Seafood, School of Food Science and Technology, Dalian Polytechnic University, Dalian 116304, China; lishugang688@163.com (S.L.); 19841133861@163.com (Q.R.); shouhao1105@163.com (S.Z.); songshuang@dlpu.edu.cn (S.S.)

**Keywords:** *Lactobacillus rhamnosus*, *Pediococcus acidilactici*, anti-inflammatory, probiotic potentials, colitis

## Abstract

Probiotics have garnered escalating attention in the treatment and prevention of inflammatory disorders. In this study, *Lactobacillus rhamnosus* RL-H3-005 (RL5) and *Pediococcus acidilactici* RP-H3-006 (RP6), which possess anti-inflammatory effects and favorable probiotic attributes, were selected through the comparison of an RAW264.7 inflammatory cell model screening and in vitro probiotic properties. Subsequently, it was implemented in an animal model of dextran sulfate sodium (DSS)-induced colitis. The results demonstrated that RL5 and RP6 could inhibit the release of proinflammatory factors in RAW264.7 inflammatory cells and exhibited excellent environmental adaptability, adhesion, safety, and antibacterial activity. Additionally, RL5 and RP6 provided protective effects on the intestines of mice with acute colitis by reducing the levels of intestinal inflammation and oxidative stress. Concurrently, supplementation with RL5 and RP6 modulated the composition of the gut microbiota in mice. These discoveries suggest that RL5 and RP6 can be used as a novel probiotic for alleviating intestinal inflammation.

## 1. Introduction

Probiotics are living microorganisms that can exert a positive influence on the host organism when consumed in adequate amounts [1]. Currently, probiotics are considered the most prominent and commonly utilized functional food product [2]. Among the numerous functions of probiotics, their immunomodulatory and anti-inflammatory properties are particularly notable [3]. Probiotics are capable of directly or indirectly modulating the host immune response and diminishing the damage induced by oxidative stress and inflammation by regulating the structure and diversity of gut microbiota and restoring the equilibrium of intestinal flora [4]. Additionally, probiotic intervention can enhance the abundance of beneficial bacteria such as *lactic acid bacteria* (LAB) and *Bifidobacterium*, thereby augmenting the generation of beneficial metabolites like short-chain fatty acids (SCFA) and facilitating the regulation of host immune function [5,6]. Nevertheless, systematic investigations into the anti-inflammatory effects of probiotics are still limited.

At present, the anti-inflammatory effect of LAB has been increasingly recognized by a growing number of studies. For instance, the anti-inflammatory effects of *L. rhamnosus* and *Lactobacillus plantarum* have been verified in both in vivo and in vitro inflammatory models [7]. Liu et al. discovered that *L. plantarum* HF05, selected from Qula, could suppress the release of proinflammatory cytokines in RAW264.7 inflammatory cells [8]. *L. brevis* IBRC-M10790 was also demonstrated to reduce intestinal inflammatory infiltration and oxidative stress induced by DSS in mice [9]. Additionally, the inactivation of probiotics or the metabolites of certain probiotics exerts a crucial role in modulating the body’s immunity and restraining excessive inflammatory responses. *L. rhamnosus* GG can inhibit periodontal inflammation through the regulation of MAPK and NF-κB signaling pathways [10]. The lysate of *L. plantarum* K8 can suppress obesity-induced inflammatory responses by regulating the NF-κB pathway [11]. Exopolysaccharides from *L. plantarum* JLAU103 can adjust the structure of the gut microbiome to alleviate intestinal inflammation [12]. However, the research regarding the modulatory and anti-inflammatory effects of probiotics remains at an early stage, and the types of probiotics possessing anti-inflammatory effects in clinical practice are rather limited. Additionally, the precise mechanism through which probiotics exert their anti-inflammatory actions still awaits further investigation.

At present, the sources available for probiotics that can be utilized to screen for anti-inflammatory effects are relatively restricted. The feces of healthy adults or infants, as well as fermented foods such as pickles, serve as sources of probiotics possessing good probiotic characteristics or anti-inflammatory capability [13,14]. LAB isolated from intestinal tracts of infants possess the capacity to endure gastric and intestinal fluids and have a greater propensity to exert anti-inflammatory effects in vivo. For instance, *Lactobacillus* originating from the infant feces can activate macrophages and lead to an augmentation in IL-10 secretion, thereby exerting an immunomodulatory effect [15]. Recently, probiotics have emerged as potential, novel, and natural therapeutic agents [16]. Thus, isolation and characterization of new strains are still required.

In this study, we identified two novel probiotic strains of *Lactobacillus rhamnosus* RL-H3-005 and *Pediococcus acidilactici* RP-H3-006 with anti-inflammatory properties. They exhibited favorable probiotic characteristics and were capable of inhibiting LPS-induced NO release as well as the production of inflammatory factors in RAW264.7 inflammatory cells. In addition, they showed protective effects in a DSS-induced colitis animal model, offering novel perspectives in the quest for new therapeutic strategies for intestinal inflammation.

## 2. Materials and Methods

### 2.1. Raw Materials and Chemical Reagents

MRS medium was purchased from Qingdao Hi-tech Industrial Park Hope Bio-technology Co., Ltd. (Qingdao, China). DSS (molecular weight of 36–50 kDa) was obtained from MP Biomedicals, LLC (Santa Ana, CA, USA). ELISA kits for TNF-α, IL-10 and IL-6 were acquired from Shanghai Enzyme-linked Biotechnology Co., Ltd. (Shanghai, China); Nitric oxide (NO) assay kits and an ROS Assay Kit with CM-H2DCFDA were obtained from Beyotime Biotechnology (Shanghai, China); Myeloperoxidase (MPO) and Catalase (CAT) assay kits were bought from Nanjing Jiancheng Biotechnology Research Institute Co., Ltd. (Nanjing, China). All chemicals and solvents used in the various analyses were of analytical grade.

### 2.2. Strain Culture

In the current study, 21 LAB strains were isolated from the feces of healthy infants and obtained from the National Engineering Research Center of Seafood at Dalian Polytechnic University. The culture conditions were as follows: 21 strains were inoculated into pre-prepared and sterilized MRS medium, followed by cultivation in a constant temperature incubator (Zhicheng, Shanghai, China) at 37 °C for 48 h.

### 2.3. Preliminary Screening of Anti-Inflammatory Probiotics

In accordance with the approach of Liu et al. [8], 21 LAB were initially screened for anti-inflammatory activity through co-culturing with LPS-induced RAW264.7 inflammatory cells. RAW264.7 cells (Saibaikang Biotechnology Co., LTD, Shanghai, China) were cultivated in a humidified atmosphere with 5% CO_2_ within DMEM high-sucrose medium (Gibco, Riverside, CA, USA) supplemented with 10% (*v*/*v*) fetal bovine serum (FBS) (PAN-Biotech, Germany) and 1% (*v*/*v*) penicillin–streptomycin (BI, Israel) at 37 ˚C. Cells were cultured for 24 h in 96-well plates with a density of 2 × 10^4^ cells/mL. The selected strains were twice washed with PBS and resuspended in DMEM devoid of antibiotics. Subsequently, the strains were inoculated into each well at a concentration of 1 × 10^9^ CFU/mL and co-cultured with the cells for 2 h, followed by incubation with LPS (1 µg/mL) for an additional 24 h [8]. The content of NO in the collected cell supernatant was determined by the Griess method (Beyotime Biotechnology, Shanghai, China). Based on the level of inhibition of NO release, strains with anti-inflammatory activity were selected and subsequently evaluated for their probiotic properties. The content of NO in the collected cell supernatant was determined by means of the Griess method (Beyotime Biotechnology, Shanghai, China). Based on the extent of inhibition of NO release, strains possessing anti-inflammatory activity were selected and thereafter evaluated for their probiotic attributes.

### 2.4. Tolerance to Low pH and Bile

The assessment of tolerance to low pH or bile salt content was conducted using the methodology reported by Liu et al. [17]. In brief, cultures incubated overnight (15 h) underwent a process of washing three times, were subsequently resuspended in a sterile solution in PBS (Solarbio, Beijing, China), and then were inoculated in the corresponding MRS medium at a final inoculum concentration of 1.0 × 10^8^ CFU/m. In the acid resistance testing, the pH of the MRS medium was modulated to 3.0, while the LAB viability at pH 7.0 served as the control. Regarding the bile salt tolerance test, 0.3% bile salt (Macklin, Shanghai, China) was incorporated into the MRS medium, and the LAB viability in the MRS without bile salts served as the control. The acid and bile tolerance tests were conducted by gauging the LAB survival after 4 h of incubation at 37 °C. The survival rate was determined through the 3-(4, 5-dimethylthiazol-2-yl)-2, 5-diphenyltetrazolium (MTT) (Sigma, Virginia Beach, VA, USA) assay. After 4 h of incubation with MTT, the optical density at 490 nm was measured.

### 2.5. Safety Evaluation

#### 2.5.1. Hemolytic Assay

The hemolysis of the strains was probed using Columbia agar (Hopebio, Qingdao, China) containing 5% sheep blood (Solarbio, Beijing, China). The isolated strains were inoculated onto blood agar plates and thereafter placed in a constant temperature incubator set at 37 °C for a period of 24–48 h. The hemolytic pattern was examined and classified by observing strain color and the presence or absence of a hemolytic ring.

#### 2.5.2. Production of Toxic Metabolites

Amino acid decarboxylase assay: the bacterial solution was inserted into lysine broth (Hopebio, Qingdao, China) and ornithine broth (Hopebio, Qingdao, China), then incubated at 37 °C for 24 h. The color change of the culture was observed. Compared with the uninoculated medium, the medium was yellow for negative and purple for positive. *Staphylococcus aureus* ATCC29213 and *Vibrio parahaemolyticus* ATCC17802 were used as positive controls for ornithine and lysine decarboxylase assays, respectively.

Nitrate reductase detection: we used a nitrate reductase detection kit (Hopebio, Qingdao, China) to detect the nitrate reduction ability of five kinds of probiotics. We inoculated 100 μL of overnight-cultured strains into a nitrate broth medium and cultured them at 37 °C for 48 h. Then, two drops of liquid A and B were added to the nitrate broth, and the color change was observed. The medium appears red as positive and non-discolored as negative. *Listeria monocytogenes* ATCC19115 was used as a positive control.

#### 2.5.3. Antibiotic Susceptibility Test

The susceptibility of probiotic isolates was assessed using the antibiotic disc diffusion method [18]. We determined the resistance of all strains to streptomycin, ampicillin, chloramphenicol, gentamicin, vancomycin, and carbenicillin using antibiotic paper slips. We measured the zone of inhibition (if present) around the paper sheet and expressed it in millimeters (mm). Antibiotic susceptibility levels were assessed according to the method of Sui et al. [19].

### 2.6. Antimicrobial Activity Assessment

Selected LAB was tested for antibacterial activity against *Escherichia coli* ATCC25922, *S. aureus* ATCC29213, and *L. monocytogenes* ATCC19115 (National Engineering Research Center of Seafood at Dalian Polytechnic University) using the spot-on-the-lawn technique adapted from Barbosa et al. [20]. In short, the three pathogenic bacteria cultivated overnight (approximately 1 × 10^8^ CFU/mL) were evenly spread on the LB agar medium, and then an Oxford cup was placed vertically on them and gently pressed so that there were no gaps. Screened LAB culture supernatant (150 μL) cultivated overnight was added to an Oxford cup and cultured upright at 37 °C for 24 h. The growth-free inhibitory zone (mm) surrounding the Oxford cups was measured to determine the antibacterial activity of the LAB.

### 2.7. Detection of Cell Surface Properties

#### 2.7.1. Surface Hydrophobicity

The hydrophobicity of selected LAB was determined by the bacterial carbon–hydrocarbon compound adhesion method [21]. The culture that had been incubated overnight was subjected to centrifugation and subsequently washed three times using sterile PBS; the OD_600_ was then adjusted to a range of 0.1–0.2, and this value was recorded as A_0_. The culture (2 mL) was combined with equal volumes of trichloromethane and xylene, subjected to vortexing for 2 min, and incubated at 37 °C for 2 h. Subsequently, the aqueous phase was removed, and the value of OD_600_ was recorded as A_t_. The hydrophobicity (%) of the strains is defined as Cell surface hydrophobicity % = [(A_0_ − A_t_)/A_0_] × 100.

#### 2.7.2. Bacterial Auto-Aggregation

Bacterial auto-aggregation was performed according to Xu et al. [22]. The LAB were washed 3 times, then resuspended in PBS. Then, the OD_600_ was adjusted to 0.1–0.2 and labeled as A_0_. The bacterial suspension was incubated (2 h, 37 °C), and the upper layer was measured for OD_600_, labeled as A_t_. The auto-aggregation percentage is defined as Agg % = [(A_0_ − A_t_)/A_0_] × 100.

#### 2.7.3. Adhesion to HT-29 Cells

We evaluated the adhesion of the selected strains in accordance with the approach proposed by Horn et al. [23]. HT-29 cells (Abundant Bio, Hunan, China) were cultivated in a humidified setting with 5% CO_2_ within RPMI-1640 medium (from Gibco, Riverside, CA, USA) supplemented with 10% (*v*/*v*) fetal bovine serum (FBS) and 1% (*v*/*v*) penicillin–streptomycin at 37 ˚C. HT-29 cells in a favorable growth state were cultured in 12-well tissue culture plates (1 × 10^6^ cells /mL). Overnight cultures of the bacteria (10^8^ CFU/mL) were washed two times using PBS and then suspended in a cell medium devoid of antibiotics (Gibco, USA). A volume of 100 μL of the strain suspension was added to each well and co-cultured with the cells for 3 h. Upon the conclusion of the incubation period, the plates were washed two times in sterile PBS. Subsequently, 1 mL of trypsin–EDTA (from Gibco, Riverside, CA, USA) was introduced to each well and they were incubated at 37 °C for 2 min. The resultant cell suspensions were successively serially diluted in sterile PBS and inoculated onto MRS agar (37 °C, 36 h), and the number of colonies was noted as X_1_ CFU/mL. The quantity of probiotics added per well was recorded as X_0_ CFU/mL. Adhesion calculation: Adhesion % = (X_1_/X_0_) × 100

### 2.8. Molecular Identification

The genomic DNA of five strains (RL-H3-002, RL-H3-005, RP-H3-006, RF-H1-012, RF-H1-014) was extracted according to the methodology used by Liu et al. [24]. 16SrRNA gene amplification was conducted by employing universal primers, namely 27F (5′-AGAGTTTGATCMTGGCTCAG-3′) and 1492R (5′-GGTTACCTTGTTACGACTT -3′). The PCR products were inspected via 1% (*w*/*v*) agarose gel electrophoresis and visualized under a UV transilluminator. The amplicons were dispatched to Sangon Biotech Co., Ltd. (Shanghai, China) for purification and sequencing. The obtained raw data were aligned to closely related species by means of the Basic Local Alignment Search Tool (BLAST) on the National Center for Biotechnology Information (NCBI, https://www.ncbi.nlm.nih.gov/, accessed on 1 January 2024).

### 2.9. Comprehensive Evaluation

According to the method of Wang et al. [25], the statistical method rank sum ratio (RSR) was employed to conduct a comprehensive assessment of the prebiotic properties of the preliminarily screened LAB. The status of the subjects, ranging from the poorest to the best, was determined through ranking or ordinal classification. LAB isolates (n) served as the subjects, and acid tolerance, bile salt tolerance, cell hydrophobicity, self-condensation rate, and adhesion ability were utilized as variables (m). Upon the establishment of the n × m matrix, rank transformation was executed. Higher values for all the variables in this research signify more favorable outcomes. SPSSPRO (https://www.spsspro.com/, accessed on 15 May 2024) was utilized for data analysis.

### 2.10. Cell Model Analysis

RAW264.7 cells were cultured in 24-well plates with a density of 1 × 10^6^ cells/mL, and were continuously cultivated at 37 °C and 5% CO_2_ for 24 h. RL5 and RL6 (1 × 10^8^ CFU/mL) were resuspended in the cell culture medium lacking antibiotics and inoculated into each well, then co-cultured with the cells for 2 h [8]. Subsequently, 1 μg/mL LPS was added to the culture for 24 h, and both the cells and the supernatants were collected for subsequent experiments.

### 2.11. DSS-Induced Colitis

Male C57BL/6J mice at the age of 8 weeks were procured from Liaoning Changsheng Biotechnology Co., Ltd. (Shenyang, Liaoning). The mice were maintained in a controlled laboratory setting where the temperature was sustained at 23 ± 2 °C, the relative humidity was regulated at 55 ± 5%, and the light/dark cycle was set at 12 h. Both water and food were supplied ad libitum to all mice. The animal experiments were carried out in compliance with the National Institutes of Health Regulations for Laboratory Animal Management and were approved by the Animal Ethics Committee of Dalian University of Technology (Ethical Review No. DLPU2024021). After one week of acclimation, the mice were randomly allocated into four groups (6 mice per group), namely: (1) mice that were treated solely with saline for 14 days (NC group); (2) mice that were treated with saline for 14 days, and 3% DSS was added to the drinking water during the last week (DSS group); (3) mice that were gavaged with 10^9^ CFU of RL5, and 3% DSS was added to the drinking water during the last week (DSS + RL5 group); (4) mice that were gavaged with 10^9^ CFU of RP6, and 3% DSS was added to the drinking water during the last week (DSS + RP6 group). The intervention dose of LAB was chosen based on the study conducted by Liu et al. [4]. Subsequently, all the mice were sacrificed on day 21. In accordance with previous reports, the Disease Activity Index (DAI) was recorded on a daily basis for each mouse throughout the DSS treatment [26]. Serum, colon tissue, and cecal contents were collected and preserved at −80 °C. A portion of the tissue from the distal colon was fixed in 4% paraformaldehyde (Solarbio, Beijing, China) for subsequent analysis.

### 2.12. Analysis of Inflammation and Oxidative Stress Levels

Cell supernatants of IL-6, IL-10, and TNF-α, as well as serum concentrations of IL-6 and TNF-α, were analyzed by means of the corresponding ELISA kits. The mRNA expression levels of IL-10, IL-6, and TNF-α in RAW264.7 cells and the mRNA expression levels of iNOS, IL-10, IL-6, and TNF-α (Table 1) in the colon were determined through a quantitative reverse transcription–polymerase chain reaction (RT-PCR) approach [27]. CAT and MPO in the colon were measured using a commercial kit. The ROS level in RAW264.7 cells was detected with the ROS Assay Kit employing CM-H2DCFDA [28].

### 2.13. Histopathology Analysis

Fixed colon tissues were embedded in paraffin and sliced into 5 μm sections for subsequent hematoxylin and eosin (H&E) staining. The tissue staining was carried out by Jinzhiyuan Biotechnology Co., LTD. (Shenyang, China). Light microscopy (Nikon Eclipse Ci, Tokyo, Japan) was employed. The inflammation within the distal colon sections was assessed by means of established methods [27].

### 2.14. Microbial Analysis

Collected mouse fecal samples were stored at −80 °C. Total genomic DNA was extracted from the fecal samples by employing the TGuide S96 Magnetic Soil/Stool DNA Kit (Tiangen Biotech (Beijing) Co., Ltd.) in accordance with the manufacturer’s instructions. The hypervariable region V3-V4 of the bacterial 16S rRNA gene was amplified using primer pairs 338F: 5′-ACTCCTACGGGAGGCAGCA-3′ and 806R: 5′-GGACTACHVGGGTWTCTT-3′. The PCR products were verified on agarose gel and purified via an Omega DNA purification kit (Omega Inc., Norcross, GA, USA). The purified PCR products were collected, and the paired ends (2 × 250 bp) were sequenced on the Illumina Novaseq 6000 platform.

### 2.15. Statistical Analysis

Statistical analyses were performed using GraphPad Prism 9.0 software (La Jolla, CA, USA). All data were expressed as mean ± standard error of the mean (SEM). The statistical significance of differences (*p* < 0.05) was determined using ANOVA and Tukey’s multiple comparison test.

## 3. Results

### 3.1. Preliminary Assessment of the Anti-Inflammatory LAB

In this study, 21 LAB strains isolated from infant feces were preliminarily screened. Among the selected strains, five strains (RL-H3-002, RL-H3-005, RP-H3-006, RF-H1-012, and RF-H1-014) were capable of significantly inhibiting the release of NO from RAW264.7 inflammatory cells (Table 2). Among them, RL-H3-002 (RL2), RL5, and RP6 exhibited better inhibitory effects (*p* < 0.01). Treatment with RL2, RL5, and RP6 decreased the levels of NO in the supernatant by 48.19%, 53.39%, and 57.65%, respectively. It is provisionally postulated that these five LAB possess anti-inflammatory activity.

### 3.2. Evaluation of the Probiotic Properties of the Five LAB

#### 3.2.1. Acid and Bile Salt Tolerance

**Figure 1:**
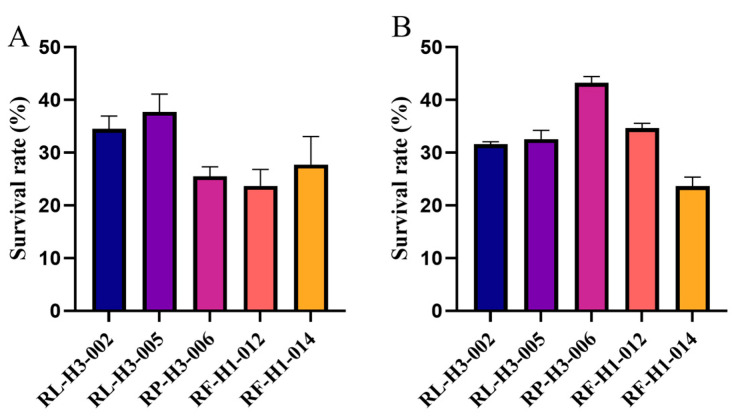
The survival rates of 5 LAB strains incubated for 4 h in MRS medium at (**A**) pH 3.0 or (**B**) 0.3% bile, *n* = 3.

Tolerance to low pH and high concentrations of bile salts constitutes an important attribute for probiotics to exert a positive impact within the host. Regarding low pH tolerance, five LAB strains could grow at pH 3.0 (Figure 1A). Among them, RL5 exhibited superior tolerance, attaining a survival rate of 37.72% after 3 h of incubation in a low-pH medium. Additionally, RP6 demonstrated the optimal tolerance to bile salts among the five isolates, with a survival rate of 43.23% (Figure 1B).

#### 3.2.2. Characterization of Safety Evaluation

Regarding the safety assessment, all five selected strains showed γ hemolysis (no hemolysis). None of the five LAB strains changed the color of the ornithine and lysine media, indicating that they did not produce putrescine and cadaverine (Table 3). Concurrently, the five LAB strains demonstrated negative nitrate reductase activity (Table 3). Furthermore, it was noted that all five strains exhibited sensitivity to at least one antibiotic (Table 3). Significantly, all isolates were susceptible to chloramphenicol, except for RP-H3-006. Additionally, all the selected strains were found to be resistant to vancomycin and gentamicin.

#### 3.2.3. Results of Antimicrobial Activity

The antimicrobial activity of the five strains isolated from infant feces was examined in opposition to *E. coli* ATCC25922, *L. monocytogenes* ATCC19115, and *S. aureus* ATCC29213. It was noted that two LAB (RF12, RF14) failed to display any antimicrobial activity against the three pathogenic bacteria (Table 4). In contrast, the remaining three strains (RL2, RL5, RP6) manifested superior antimicrobial activity towards *S. aureus* ATCC29213 and *L. monocytogenes* ATCC19115. Significantly, none of the LAB exhibited inhibition against *E. coli* ATCC25922.

#### 3.2.4. Preliminary Assessment of Bacterial Surface Characterization

**Figure 2:**
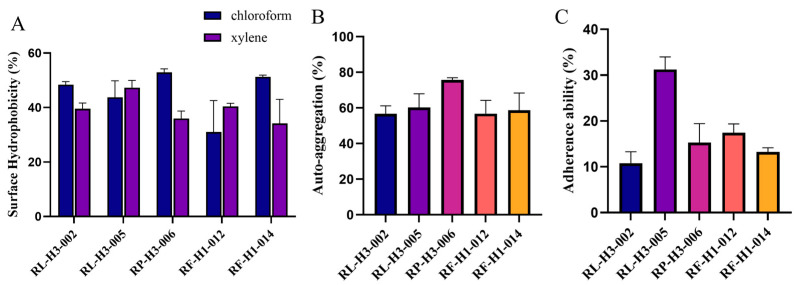
Results of surface characterization of 5 LAB. (**A**) Surface hydrophobicity, (**B**) auto-aggregation rate, (**C**) adherence ability of LAB to human HT-29 cells, *n* = 3.

Figure 2A shows the diverse degrees of hydrophobicity of LAB to xylene and chloroform. RL5 exhibited a hydrophobicity of 47.31% towards xylene, while the remaining strains displayed hydrophobicity ranging from 34.20% to 40.34%. In terms of chloroform, RP6 demonstrated the highest hydrophobicity at 52.94%. The strain with the highest auto-aggregation rate was RP6 at 75.68% (Figure 2B). The cell adherence ability disclosed varying magnitudes of adhesion to HT-29 cells among the five strains. RL5 exhibited the highest percentage of adhesion at 31.25%, while the other strains displayed adhesion percentages ranging from 10.77% to 17.45% (Figure 2C).

#### 3.2.5. Comprehensive Analysis

The selected LAB were comprehensively assessed with the aforementioned quantifiable probiotic indices. The five LAB strains were ranked in accordance with their probiotic potential by computing the RSR regression values (Table 5). As all the tested attributes were positive in the metrics, a strain with a lower RSR ranking was favored. Based on this result and the results of safety and antimicrobial activity, two isolates, namely RL5 and RP6, were selected for further study.

### 3.3. RL5 and RP6 Inhibited LPS-Induced Inflammation and Oxidative Damage in RAW264.7 Cells

**Figure 3:**
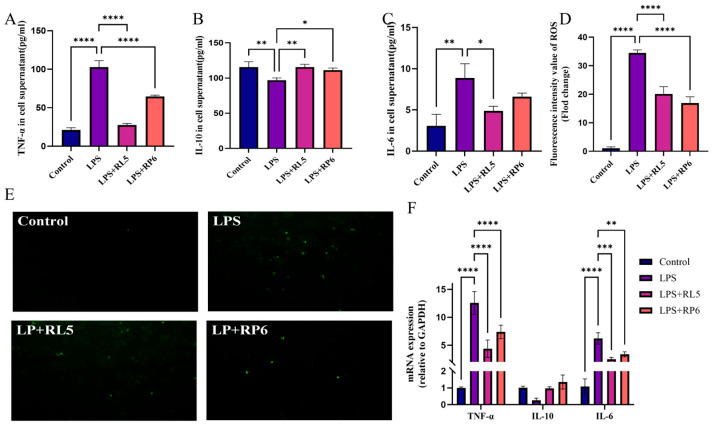
RL5 and RP6 inhibited LPS-induced inflammation and oxidative damage in RAW264.7 cells. Cell supernatants were measured for (**A**) TNF-α, (**B**) IL-10, and (**C**) IL-6 by ELISA. (**D**) Fluorescence intensity value of ROS. (**E**) Representative image of ROS (green fluorescence). (**F**) mRNA expression levels of TNF-α, IL-10, and IL-6. *n* = 3. * *p* < 0.05, ** *p* < 0.01, *** *p* < 0.001, **** *p* < 0.0001.

Exposure of RAW264.7 cells to LPS for 24 h led to a significant increase in the secretion of the proinflammatory cytokines IL-6 (*p* < 0.01) and TNF-α (*p* < 0.0001), while the release of the anti-inflammatory cytokine IL-10 (*p* < 0.01) from RAW264.7 cells was significantly reduced. Nevertheless, the intervention of RL5 and RP6 significantly mitigated this tendency (Figure 3A–C). Moreover, the mRNA expression levels of IL-6, IL-10, and TNF-α were evaluated (Figure 3F). The results indicated that the mRNA expression levels of IL-6 and TNF-α were prominently enhanced (*p* < 0.0001). Treatment with RL5 and RP6 significantly suppressed the alterations in the mRNA levels of these genes (*p* < 0.001). Additionally, it was observed that ROS production was relatively downregulated in RAW264.7 cells pretreated with RL5 and RP6 compared to the LPS group (Figure 3E). These findings implied that RL5 and RP6 alleviated the LPS-induced inflammatory response and oxidative stress in RAW264.7 cells and demonstrated excellent anti-inflammatory activity in vitro.

### 3.4. Oral Administration of RL5 and RP6 Improved the Symptoms of Experimental Colitis

**Figure 4:**
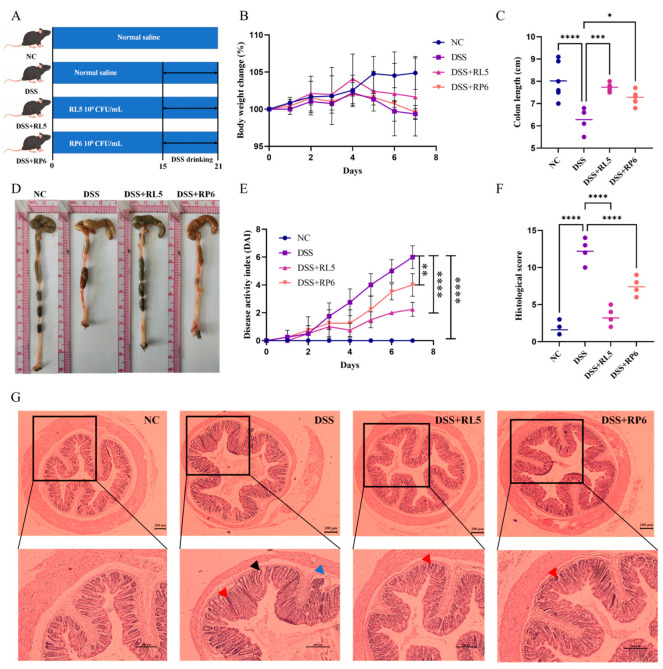
Oral administration of RL5 and RP6 improved the symptoms of experimental colitis. (**A**) Experimental design. (**B**) Percent of body weight loss in mice during free drinking of 3% DSS water. (**C**) Lengths of the colon and (**D**) representative images of each group of mice. (**E**) Disease activity index (DAI) of each group of mice. (**F**) Histological scores of colon sections. (**G**) Microphotographs of H&E-stained sections of colons (upper row, 40×; lower row, 100×; scale bar = 100 μm). Arrowhead inflammatory cell infiltrates within mucosa (red) and submucosa (blue); black arrowhead, goblet cell loss. All data are presented as mean ± SEM. *n* = 4–6. * *p* < 0.05, ** *p* < 0.01, *** *p* < 0.001, **** *p* < 0.0001.

DSS-induced mice were used as a colitis model for evaluating the in vivo anti-inflammatory effects of RL5 and RP6 (Figure 4A). As shown in Figure 4B, upon 7 days of DSS treatment, mice in the DSS group exhibited a remarkable decrease in body weight. The intervention of RL5 improved the body weight loss of mice, while RL6 failed to improve the body weight loss situation. Additionally, oral administration of RL5 and RL6 significantly decreased the DAI scores (*p* < 0.01) (Figure 4E). The colons of DSS group mice were significantly shortened and showed congestion and edema (*p* < 0.0001, Figure 4C,E), while RL5 and RL6 could conspicuously prevent the colon shortening induced by DSS (*p* < 0.05). Histopathology analysis of the colon showed that the intake of DSS damaged the colon tissue of mice, leading to an augmented number of inflammatory cell infiltrations in the submucosa and a decreased thickness of the colon wall (Figure 4G). However, supplementation with RL5 and RP6 significantly improved DSS-induced colonic pathological changes and reduced histological scores (*p* < 0.0001).

### 3.5. Oral Administration of RL5 and RP6 Regulated Proinflammatory Cytokine Secretion and Oxidative Stress in DSS-Induced Colitis Mice

**Figure 5:**
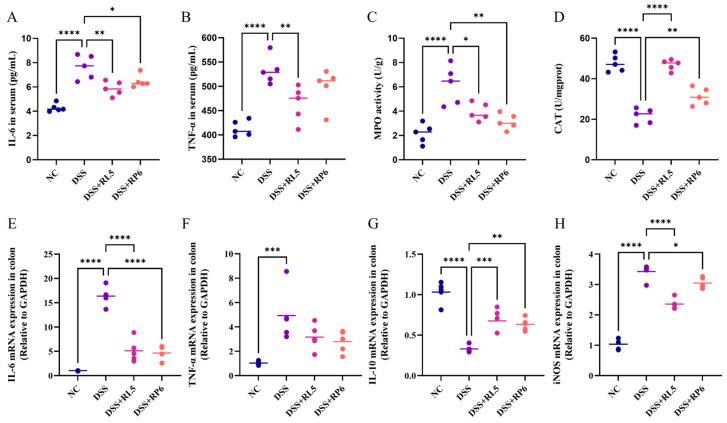
Oral administration of RL5 and RP6 regulated proinflammatory cytokine secretion and oxidative stress in DSS-induced colitis mice. (**A**) Serum was measured for (**A**) IL-6 and (**B**) TNF-α by ELISA. (**C**) MPO activity. (**D**) CAT level. mRNA expression levels of (**E**) IL-6, (**F**) TNF-α, (**G**) IL-10, and (**H**) iNOS. All data are presented as mean ± SEM. *n* = 4–6. * *p* < 0.05, ** *p* < 0.01, *** *p* < 0.001, **** *p* < 0.0001 between groups.

To assess the degree of neutrophil infiltration, the MPO activity within colon tissue was determined. As shown in Figure 5C, supplementation with RL5 (*p* < 0.05) and RP6 (*p* < 0.01) significantly reduced MPO activity compared with the DSS group. The overproduction of proinflammatory factors aggravates the progression of colitis. In the current study, the serum levels of IL-6 and TNF-α were prominently increased in DSS-induced colitis mice (*p* < 0.0001), which were significantly decreased after RL5 supplementation (*p* < 0.01), but RP6 did not significantly inhibit the alterations of TNF-α (Figure 5A,B). In addition, the mRNA expression levels of cytokines in colon tissues were measured (Figure 5E–H). We found that RL5 and RP6 intervention significantly reduced the mRNA expression level of IL-6 (*p* < 0.0001) and increased the expression level of IL-10 (*p* < 0.01) in the colon. Concurrently, the supplementation of RL5 and RP6 also significantly enhanced the level of CAT (*p* < 0.01) (Figure 5D) and the mRNA expression level of iNOS in colon tissues (*p* < 0.05).

### 3.6. Oral Administration of RL5 and RP6 Regulates the Gut Microbiota in DSS-Induced Colitis Mice

**Figure 6:**
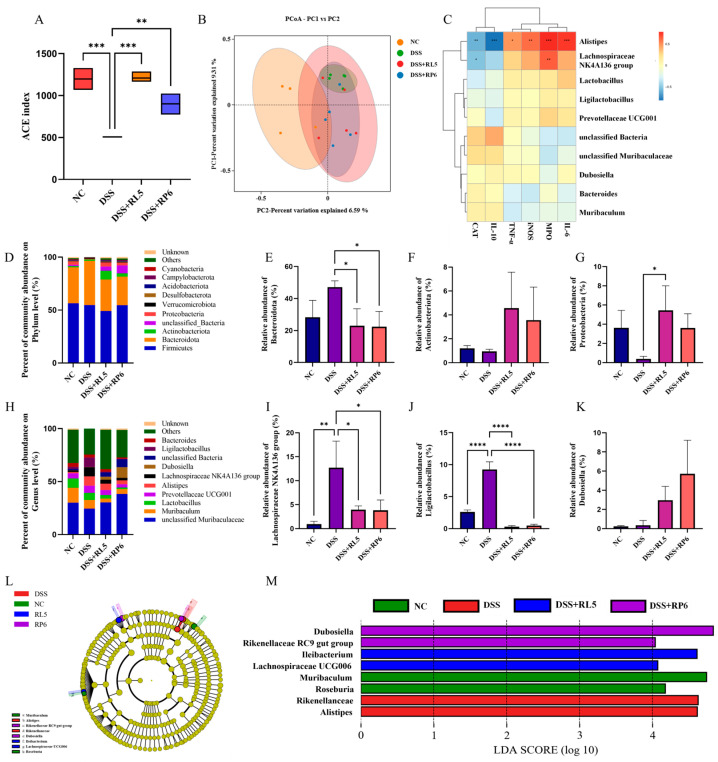
Oral administration of RL5 and RP6 regulates the gut microbiota in DSS-induced colitis mice. (**A**) ACE index; (**B**) PCoA of the β-diversity of microbial composition in mice; (**C**) correlation analysis of specific gut bacteria and inflammatory factors between 4 groups. (**D**–**G**) Relative abundance (%) at the phylum level; (**H**–**K**) relative abundance (%) at the genus level; (**L**–**M**) Linear Discriminant Analysis (LDA) indicates the most differential abundant bacterial taxa in each sample. The data were expressed as mean ± SEM. *n* = 4–5. * *p* < 0.05, ** *p* < 0.01, *** *p* < 0.001, **** *p* < 0.0001 between groups.

The alpha diversity index was employed to appraise the alterations in microbial communities among the groups. The intervention of RL5 and RP6 led to a remarkable increase in the ACE index in comparison with the DSS group (*p* < 0.01) (Figure 6A). Principal coordinate analysis (PCoA) was utilized to assess the disparities in microbial composition among the groups (Figure 6B). The findings demonstrated that the microbial community was significantly modified in the DSS group, and this was partially reversed by the supplementation of RL5 and RP6. Additionally, we conducted an analysis of the relative abundance of gut microbiota. At the phylum level (Figure 6D), DSS treatment diminished the relative abundance of Firmicutes while enhancing that of Bacteroides. The supplementation of RL5 and RP6 effectively reversed the disordered ratio of Firmicutes to Bacteroidetes (Figure 6E). The RL5 intervention also augmented the relative abundance of Actinobacteriota and Proteobacteria (Figure 6F,G). At the genus level (Figure 6H), the relative abundance of the *Lachnospiraceae* NK4A136 group and *Ligilactobacillus* was significantly elevated in the DSS group, a tendency that was altered by both the RL5 and RP6 interventions (Figure 6I,J). Notably, supplementation with RL5 and RP6 resulted in a large increase in the relative abundance of *Dubosiella* (Figure 6K). To further illustrate the major differences in OTUs among the five groups, we employed LEfSe (Linear Discriminant Analysis (LDA) Effect Size) (Figure 6L,M). DSS treatment significantly altered the dominant microbiota compared to the NC group, particularly by increasing the relative abundance of *Rikenellanceae* and *Alistipes*. In contrast, LAB supplementation was able to reduce the relative abundance of these dominant microbiota by enriching another microbiota. For example, *Ileibacterium* increased in the OVA/L5 group, while OVA/L6 increased the relative abundance of *Dubosiella*. To further elucidate the impact of LAB on gut microbiota, we selected bacteria from the top 10 in terms of relative abundance in four groups for correlation analyses with inflammatory markers (Figure 6C), including MPO, IL-6, IL-10, TNF-α, iNOS, and CAT (Figure 6H). Our findings revealed that 12 bacteria exhibited significant positive or negative correlations with at least one parameter of allergic asthma following supplementation with different LAB strains. Notably, the relative abundance of *Alistipes* showed positive correlations with MPO, IL-6, TNF-α, and iNOS, but was negatively correlated with IL-10 and CAT. In addition, the *Lachnospiraceae* NK4A136 group was also significantly positively correlated with MPO and negatively correlated with CAT.

## 4. Discussions

LAB are the most extensively investigated microorganisms in the mechanism of action and utilization efficiency of probiotics, and they can serve as biotherapeutic or prophylactic agents [29]. Recently, more attention has been paid to the study of how LAB regulate innate and adaptive defense responses and maintain intestinal homeostasis [8,30]. In this study, we selected two strains with anti-inflammatory potential, RL5 and RP6, by assessing the impact of the strains on the release of NO in inflammatory RAW264.7 cells and their in vitro probiotic characteristics. Most significantly, their protective effects in chronic inflammatory bowel disease were examined in an animal model. In comparison to other inducers, DSS can disrupt intestinal epithelial cells in mice and induce inflammatory responses, which bear significant resemblance to human symptoms [27]. Hence, the DSS-induced enteritis model was opted for to conduct a comprehensive assessment of the probiotic characteristics and anti-inflammatory potential of RL5 and RP6.

The screening of novel functional probiotics typically encompasses the assessment of various properties, such as resistance to digestive enzymes, bile, and acids, adhesion to mucus or intestinal epithelial cells, interaction with human immune cells, and antimicrobial activity [31]. LAB derived from the infant gut are more conducive to the human environment and are regarded as ideal contenders for the development of novel potential probiotics [32]. In our study, both RL5 and RL6 showed excellent tolerance to bile or acid, which is in accordance with the previous findings that the majority of lactic acid bacteria possess superior environmental tolerance [33]. Within a certain range, the increase in the intake of probiotics can improve their concentration rate in the intestine, so as to better play the role of probiotics. Compared with other bacteria, LAB have higher safety [34]. However, due to several health incidents, safety evaluation of novel strains is requisite [35]. In our study, the selected strains, RL5 and RP6, were nonhemolytic, did not produce harmful metabolites, and were susceptible to at least one antibiotic. It should be emphasized that the phenotypic examination in our study did not encompass all the criteria for the evaluation of probiotic properties and safety. Nevertheless, it is of significant value for the initial screening of novel functional probiotic strains. The capacity of strains to adhere and compete with pathogenic bacteria is a crucial property for exerting their probiotic effects within the host [36]. In our study, the adhesion rates of RL5 and RP6 to HT-29 were 31.25% ± 4.36% and 15.29% ± 7.00%. This finding is consistent with the previously published data by Collado et al. [37], whose adhesion values ranged from 0.9% (*P. freudenreichii* JS) to 20% (*L. rhamnosus* GG). At the same time, RL5 and RP6 showed good inhibitory effects on the growth of *L. monocytogenes* and *S.aureus*. These properties might potentially serve as a crucial factor accounting for the ability of RL5 and RP6 to suppress the progression of DSS-induced colitis in mice. Nevertheless, the reason why RL5 and RP6 strains demonstrate discrepant levels of prebiotic capacity remains to be clarified. Further experiments, including whole genome sequencing analysis, will furnish more data to tackle this query.

Inflammatory cytokines are signaling molecules involved in the differentiation and development of immune cells and the regulation of immune response [38]. Excessive expression of proinflammatory cytokines frequently gives rise to systemic inflammation and tissue damage, thereby exacerbating the progression of the disease [39]. An increasing body of studies has demonstrated that a notable elevation in proinflammatory cytokines, such as IL-6, TNF-α, and IL-1β, constitutes the primary characteristic of colonic injury during the course of colitis development [28,40]. In this study, we evaluated the in vivo anti-inflammatory effects of RL5 and RP6 using various forms, including histological analysis as well as detection of inflammatory factor levels. As shown in our results, the levels of proinflammatory factors in serum and colons were significantly increased in DSS-treated mice. However, this trend was inhibited by RL5 supplementation, but the inhibitory effect of RP6 on TNF-α was not obvious. *Lactiplantibacillus plantarum* HF05 could reduce the release of TNF-α and IL-6 from LPS-treated macrophages and the levels of TNF-α and IL-6 in DSS-induced colitis mice, thereby playing an anti-inflammatory role [4]. Additionally, we also discovered that supplementation with RL5 and RP6 significantly enhanced the expression of IL-10 in the colon. IL-10, which is secreted by M2 macrophages, is predominantly engaged in anti-inflammatory processes and the repair of injured tissues [15]. *Lactococcus lactis* LB 1022 increased IL-10 levels both in vitro and in mice with atopic dermatitis [41]. Concurrently, the overexpression of proinflammatory factors activates neutrophils and macrophages, which generate oxidative stress and ultimately intensify the inflammatory response [42]. MPO is frequently employed as a marker for neutrophil infiltration and a facilitator of oxidative stress, thereby inducing tissue damage and inflammation [27]. In accordance with prior studies, MPO activity was conspicuously augmented in the DSS-induced mouse colon tissues, whereas intervention with RL5 and RP6 significantly diminished MPO activity. In addition, RL5 and RP6 supplementation also reduced the mRNA expression level of iNOS and the level of CAT in the colon. These results suggested that RL5 and RP6 exerted anti-inflammatory effects in vivo by reducing the release of proinflammatory factors and oxidative stress levels, and similar results were obtained in the RAW264.7 inflammatory cell.

The gut microbiota may exert an influence on the metabolic homeostasis of the host. Disrupted gut microbiota can give rise to a multiplicity of common inflammatory disorders [43]. Certain studies have revealed that the diversity of intestinal microbiota is diminished, and the composition of the microbiota is altered in animal models of inflammatory bowel disease (IBD) [44]. As a significant functional food, probiotics contribute to maintaining and re-establishing the normal microbial balance (homeostasis) of the gastrointestinal tract. For instance, *L. plantarum* HF05 and *L. gasseri* JM1 are capable of regulating DSS-induced disorders of the gut microbiota [8,45]. In our research, RL5 and RP6 impacted the composition of the gut microbiota, reversing the reduction in gut microbial diversity resulting from DSS treatment. Firmicutes and Bacteroidetes are the two most crucial phyla in the intestinal tracts of mice and humans [46]. The reduction in the proportion of Firmicutes and Bacteroidetes is regarded as an indicator of intestinal dysbiosis, which is associated with inflammatory bowel disease, depression, Alzheimer’s disease, etc. [28]. Just as in the previous study, DSS treatment decreased F/B, while pretreatment with RL5 and RP6 reversed this tendency. Significantly, the RL5 and RP6 interventions markedly enhanced the relative abundance of *Dubosiella*. As a vigorous SCFAs-producing commensal bacterium, *Dubosiella* is considered to possess a crucial immunomodulatory function in the maintenance of host intestinal homeostasis. Zhang et al. discovered that *Dubosiella* could activate the AhR-IDO1-Kyn pathway in dendritic cells, improve the imbalanced Treg/Th17 response, and play a role in alleviating mucosal and systemic inflammation in DSS-induced colitis [47]. Hence, RL5 and RP6 might exert their anti-inflammatory effects through enriching *Dubosiella* and increasing the production of beneficial metabolites, such as SCFAs. Nevertheless, a limitation of this study is that the metabolite level changes in RL5 and RP6 in DSS-induced colitis mice were not examined, which precludes a definite comparative study of the mechanisms by which RL5 and RP6 exert their anti-colitis effects. In addition, only a DSS-induced colitis model was used to investigate the anti-inflammatory ability in vivo, which could be applied to more in vivo inflammation models in future studies.

## 5. Conclusions

In conclusion, the combination of in vitro and in vivo studies demonstrated that *Lactobacillus rhamnosus* RL-H3-005 and *Pedicoccus acidilactici* RP-H3-006 possess excellent anti-inflammatory activity and probiotic properties. RL5 and RP6 significantly inhibited the release of inflammatory factors from RAW264.7 cells. In addition, prophylactic supplementation with RL5 and RP6 significantly alleviated the symptoms of DSS-induced colitis in mice and regulated the levels of intestinal inflammatory infiltration, proinflammatory factors, and oxidative stress, thereby exerting anti-inflammatory effects. In addition, they regulated the composition of the gut microbiota. These results suggest that RL5 and RP6 have anti-inflammatory potential and further confirm their novelty and potential implications for treating inflammatory bowel diseases., while more in vivo and even ex vivo and clinical trials are necessary.

## Figures and Tables

**Table 1:** Primer sequences (5′–3′) used for qRT-PCR.

Gene	Forward Sequence	Reverse Sequence
IL-10	GGTTGCCAAGCCTTATCGGA	GAGAAATCGATGACAGCGCC
IL-6	CTCAGCGCTGAGTTG	CCTGTAGCCCACGTCGTAGC
TNF-α	CGGGCAGGTCTACTTTGGAG	ACCCTGAGCCATAATCCCCT
iNOS	TGAGTTCCGAAGCAAGCCAA	AGACCTCAACAGAGCCCTCA
GAPDH	TGTGTCCGTCGTGGATCTGA	TTGCTGTTGAAGTCGCAGGAG

**Table 2:** NO in the supernatant of RAW264.7 inflammatory cells.

	Groups	NO (μmol/L)
1	Control	5.31 ± 0.05
2	LPS	43.83 ± 0.31
3	LPS+RL-H3-001	38.32 ± 0.12
4	LPS+RL-H3-002	22.71 ± 0.92 **
5	LPS+RL-H3-004	33.89 ± 1.21
6	LPS+RL-H3-005	20.43 ± 0.21 **
7	LPS+RL-H3-006	46.83 ± 0.01
8	LPS+RL-H3-010	37.83 ± 1.12
9	LPS+RP-H3-002	39.83 ± 0.24
10	LPS+RP-H3-003	56.83 ± 0.26
11	LPS+RP-H3-004	36.83 ± 0.09
12	LPS+RP-H3-006	18.56 ± 0.48 **
13	LPS+RP-H3-008	40.22 ± 0.89
14	LPS+RP-H3-009	40.98 ± 0.33
15	LPS+RP-H3-012	47.93 ± 0.52
16	LPS+RP-H3-014	39.02 ± 0.01
17	LPS+RP-H3-017	41.83 ± 1.81
18	LPS+RP-H3-018	52.39 ± 1.71
19	LPS+RF-H1-005	44.76 ± 0.76
20	LPS+RF-H1-009	49.03 ± 0.01
21	LPS+RF-H1-010	58.83 ± 0.30
22	LPS+RF-H1-012	31.93 ± 0.29 *
23	LPS+RF-H1-014	30.47 ± 0.01 *

All data are expressed as mean ± SEM. *n* = 3, * *p* < 0.05, ** *p* < 0.01.

**Table 3:** Results of hemolysis tests, production of toxic metabolites, and antibiotic susceptibility.

Strain	Hemolytic Activity ^a^	Production of Toxic Metabolites ^b^			Antibiotic Susceptibility ^c^					
		LD	OD	NR	AMP	C	VA	GEN	SM	CAR
RL-H3-002	γ	-	-	-	S	S	R	R	R	R
RL-H3-005	γ	-	-	-	S	S	R	R	S	R
RP-H3-006	γ	-	-	-	S	R	R	R	S	S
RF-H1-012	γ	-	-	-	R	S	R	R	R	S
RF-H1-014	γ	-	-	-	R	S	R	R	R	S

^a^ γ: gamma hemolysis (no hemolysis); ^b^ LD: lysine decarboxylation, OD: ornithine decarboxylation, NR: nitrate reductase; ^c^ AMP: ampicillin, C: chloramphenicol, VA: vancomycin, GEN: gentamicin, SM: streptomycin, CAR: carbenicillin; S: susceptible(>20 mm), R: resistance (<14 mm).

**Table 4:** Inhibitory effects of five candidate strains on pathogen growth.

Strain	*E. coli* ATCC 25922	*S. aureus* ATCC 29213	*L. monocytogenes* ATCC 19115
RL-H3-002	-	22.4 ± 1.0	28.9 ± 0.6
RL-H3-005	-	23.1 ± 1.7	26.3 ± 0.8
RP-H3-006	-	17.0 ± 0.9	24.9 ± 1.1
RF-H1-012	-	-	7.2 ± 1.3
RF-H1-014	-	-	-

All data are expressed as mean ± SEM (*n* = 3).

**Table 5:** Inhibitory effects of five candidate strains on pathogen growth.

Isolate	RSR Rank ^a^	Probit	RSR Regression	Species	GenBank
RL-H3-005	4	6.64	0.73	*Lactobacillus rhamnosus*	OR743464
RP-H3-006	4	5.84	0.59	*Pediococcus acidilactici*	OR743466
RL-H3-002	3	5.25	0.49	*Lactobacillus rhamnosus*	OR743465
RF-H1-012	3	4.74	0.40	*Enterococcus faecalis*	OR743467
RF-H1-014	2	4.16	0.29	*Enterococcus faecalis*	OR743468

^a^ Strains with lower RSR rankings are preferable.

## Data Availability

The original contributions presented in the study are included in the article; further inquiries can be directed to the corresponding author.
